# Parent and Clinician Perspectives on Challenging Parent-Clinician Relationships in Pediatric Oncology

**DOI:** 10.1001/jamanetworkopen.2021.32138

**Published:** 2021-11-17

**Authors:** Jennifer W. Mack, Tim Jaung, Hajime Uno, Julienne Brackett

**Affiliations:** 1 Department of Pediatric Oncology, Dana-Farber Cancer Institute, Boston, Massachusetts; 2 Division of Population Sciences Center for Outcomes and Policy Research, Dana-Farber Cancer Institute, Boston, Massachusetts; 3 Division of Pediatric Hematology/Oncology, Boston Children’s Hospital, Boston, Massachusetts; 4 Department of Pediatrics, Section of Pediatric Hematology/Oncology, Texas Children’s Hospital, Houston

## Abstract

**Importance:**

Parents of children with cancer value strong therapeutic relationships with oncology clinicians, but not every relationship is positive.

**Objective:**

To identify the prevalence of challenging parent-clinician relationships in pediatric oncology and factors associated with these challenges from parent and clinician perspectives.

**Design, Setting, and Participants:**

This survey was conducted among parents and oncology clinicians of children with cancer within 3 months of diagnosis from November 2015 to July 2019 at Dana-Farber Cancer Institute/Boston Children’s Hospital and Texas Children’s Hospital. Participants were 400 parents of children with cancer and 80 clinicians (ie, oncology physicians and nurse practitioners). Parents completed surveys about relationships with 1 to 2 primary oncology clinicians; clinicians completed surveys about relationships with parents. Data were analyzed from July 2020 to August 2021.

**Exposures:**

At least 3 previous clinical visits between parent and clinician.

**Main Outcomes and Measures:**

The Relationship Challenges Scale Parent Version and Clinician Version were developed and used to measure threats to the therapeutic alliance. For the Relationship Challenges Scale–Parent version, relationships were considered challenging if a parent responded to any single question in the 2 lowest of 4 possible categories. For the Relationship Challenges Scale–Clinician version, challenges were considered to be present if a clinician reported responses in the 3 lowest of 6 possible response categories to any question.

**Results:**

Among 400 parents, there were 298 [74.5%] women, 25 Asian individuals (6.3%), 28 Black individuals (7.0%), 97 Hispanic individuals (24.3%), 223 White individuals (55.8%), and 10 individuals (2.4%) with other race or ethnicity; race and ethnicity data were missing for 17 (4.3%) individuals. Among 80 clinicians, there were 57 (71.3%) women, 38 attending physicians (47.5%), 32 fellows (40.0%), and 10 nurse practitioners (12.5%). Parents identified 676 unique relationships with clinicians, and clinician reports were available for 338 relationships. Among 338 relationships with paired parent and clinician surveys, 81 relationships (24.0%) were considered challenging by parents, 127 relationships (37.6%) were considered challenging by clinicians, and 33 relationships (9.8%) were considered challenging by parent and clinician. Parents with Asian or other race or ethnicity (odds ratio [OR] vs White parents, 3.62; 95% CI, 1.59-8.26) or who had lower educational attainment (OR for ≤high school vs >high school, 3.03; 95% CI, 1.56-5.90) were more likely to experience relationships as challenging. Clinicians used a variety of strategies more frequently in 127 relationships in which they perceived challenges vs 211 relationships in which they did not perceive challenges, such as holding regular family meetings (22 relationships [17.3%] vs 13 relationships [6.2%]; *P* = .009) and offering extra time and attention (66 relationships [52%] vs 60 relationships [28.4%]; *P* < .001). However, these strategies were not used with increased frequency when parents experienced relationships as challenging vs when parents did not experience this.

**Conclusions and Relevance:**

This survey study found that nearly one-quarter of parents of children with cancer reported challenges in the therapeutic relationship with their oncologist and that clinicians used strategies to improve relationships more frequently when they experienced the relationship as challenging. These findings suggest that new strategies are needed to improve experiences for parents and to help clinicians recognize and attend to parents whose experiences are suboptimal.

## Key Points

**Question:**

How often do parents and clinicians experience challenges in the therapeutic relationship in pediatric oncology?

**Findings:**

In this survey study of 400 parents of children with cancer and 80 clinicians, 24.0% of relationships with the child’s primary oncology clinician were experienced as challenging by parents. Among clinicians, 37.6% of these relationships were experienced as challenging, but parent and clinician perspectives infrequently aligned, with 9.8% of relationships perceived as challenging by parents and clinicians.

**Meaning:**

These findings suggest that many parents experience threats to the therapeutic alliance but the challenges parents experience often go unrecognized by clinicians.

## Teaser

This survey study investigates the prevalence of challenging parent-clinician relationships in pediatric oncology and factors associated with these challenges.

## Introduction

Patient-centered care is the standard for cancer care,^1^ associated with a better experience for patients and serving as the foundation of high-quality, safe medical care.^1,2,3^ One aspect of such care is the patient-clinician relationship; patients want to be treated with sensitivity and caring, be involved in decisions about care, and have relationships based on mutual respect and trust.^4,5,6,7^ However, research in adults has found that up to 30% of patient-physician relationships are challenging,^8,9,10^ suggesting that not every patient benefits from positive relationships with clinicians.

Research on the parent-clinician relationship in pediatrics, a field in which parents rather than patients form primary decision-making relationships with clinicians, is limited.^11^ However, understanding challenges may offer opportunities to prevent or resolve difficulties and strengthen the therapeutic relationship, which extends for years in chronic pediatric conditions like cancer, crossing numerous clinical encounters across the care trajectory.

We previously conducted qualitative work to define the nature of challenges in parent-clinician relationships in pediatric oncology.^11^ We found that parents experience challenges as fractures in the therapeutic relationship, which encompasses several issues, such as listening, mutual respect, and trust. In contrast, clinicians experience challenges as relationships in which significant time and effort is required to ensure good communication and care.

Building on these definitions, we used parent and clinician surveys at 2 academic cancer centers to investigate the prevalence of challenging parent-clinician relationships in pediatric oncology, focusing on the early relationship that develops soon after diagnosis, and defining challenges as persistent issues across multiple visits rather than single negative interactions. Paired surveys were used to compare parent and clinician perspectives, identify factors associated with challenging relationships, and identify strategies that clinicians use to manage or repair these issues.

## Methods

This survey study was conducted among parents and oncology clinicians of children with cancer at Dana-Farber Cancer Institute/Boston Children’s Hospital and Texas Children’s Hospital from November 2015 to July 2019. Institutional review boards of both sites approved this study. The Dana-Farber Cancer Institute institutional review board, with agreement from the Texas Children's Hospital institutional review board, determined that documentation of informed consent was not required because this was considered a minimal risk study. This study followed the American Association for Public Opinion Research (AAPOR) reporting guideline.

One parent per child was eligible if he or she could read English or Spanish, the child was age younger than 18 years, the child had had at least 3 visits with the primary oncology clinician and was 3 months or less from diagnosis at first contact, and the primary oncology clinician gave permission for contact. Eligible parents were contacted in person or by mail and given a letter inviting them to participate, along with the survey, a postage-paid opt-out postcard, and a $10 gift card. The study was framed as a study about communication between parents and clinicians with a goal of understanding what helps communication go well and what makes it challenging. Questionnaires were available in paper and pencil and electronic format and in English and Spanish. For nonrespondents, 2 subsequent contacts were made in person or by mail or phone. Child age and cancer type were identified using medical records.

The survey asked parents to name up to 2 primary oncology clinicians involved in the child’s care based on sites’ care systems, in which an attending and fellow or nurse practitioner often work together in this role. Parents were asked to answer separate questions about each relationship. After the parent survey was complete, clinicians named by the parent were given the clinician survey, which asked about the relationship with the parent, with a $5 gift card.

### Parent Survey

#### Primary Outcome Measure

The Relationship Challenges Scale–Parent version, developed for this study, drew on questions from the Human Connection Scale for adults with advanced cancer as well as themes from our previous qualitative interviews with parents.^11,12^ Cognitive interviews with 10 parents of children with cancer were done to confirm conceptual, face, and content validity. The scale included 11 questions with responses in Likert format (eAppendix 1 in the Supplement) centered on listening, sensitivity, mutual respect, and trust. Cronbach α was 0.87. Item total correlations ranged from 0.48 to 0.75. Test-retest reliability was 0.90 for 20 relationships over a 2-day to 10-day interval. Scores were associated with parent reports of the quality of oncologist communication (mean [SD] quality of communication score, 35.2 [1.3] for relationships without challenges and 30.9 [4.6] for relationships with challenges; *P* < .001) and information (mean [SD] quality of information score, 14.9 [1.3] for relationships without challenges and 13.6 [1.8] for relationships with challenges; *P* < .001),^13,14^ supporting convergent validity.

#### Parent Attributes

The parent questionnaire asked about parent sex, race and ethnicity, educational level, and primary language. Race and ethnicity response options in the survey were Asian/Pacific Islander (hereafter, Asian), Black, Hispanic/Latino (hereafter, Hispanic), Native American, White, and other race and ethnicity (given the small number in this category, these are not further defined to protect identities). Race and ethnicity were assessed as factors hypothesized to be associated with relationship experiences given our prior work finding disparities in communication experiences in pediatric oncology. The Hospital Anxiety and Depression Scale (HADS) was used to evaluate depression and anxiety, with scores of 8 or more on each subscale considered suggestive of the condition.^15^ The HADS has 14 items, with 7 items each for anxiety and depression subscales. Scoring for each item ranges from 0 to 3, which are then summed for each subscale.

#### Systems of Care

Parents were asked how well other doctors and nurses involved in their child’s care work together (interdisciplinary teamwork), how often they are told different things by different people (mixed messages), how often different doctors and nurses caring for the child are aware of the child’s medical history (communication across transitions), and how often different doctors and nurses caring for the child are aware of his or her special needs (patient-centeredness across transitions).^16^ Response options were always, often, sometimes, rarely, and never.

### Clinician Survey

#### Primary Outcome Measure

The Relationship Challenges Scale–Clinician version, developed for this study, drew on existing questions from the Difficult Doctor-Patient Relationship Questionnaire, as well as themes from our previous qualitative interviews with clinicians.^8,9,11,17^ Cognitive interviews with 10 pediatric oncology clinicians were done to confirm conceptual, face, and content validity. The scale included 6 questions with responses in Likert format (eAppendix 2 in the Supplement), focused on challenges communicating and delivering best care. Clinician questions explicitly named challenges, in contrast with the parent scale, which focused on more positive attributes, such as listening and sensitivity. A 6-point Likert scale ranged from not at all to a great deal. Cronbach α was 0.82. Item total correlations ranged from 0.47 to 0.88. Clinicians completed the scale about each parent who had named the clinician as a primary oncology clinician in the parent survey.

#### Clinician Attributes and Strategies Used to Work With Parents

The survey asked about clinician role (ie, attending physician, fellow, or nurse practitioner), sex, race and ethnicity, and years in practice. Race and ethnicity survey response options were the same as in the parent survey. Clinicians were asked whether they had used strategies to work with the parent, including holding family meetings, apologizing, setting limits, adapting to the parent’s communication style, devoting extra time, and seeking help from other clinicians.

#### Potential Outcomes of Challenges

Clinician burnout was assessed using an item from the Maslach burnout inventory (a single-item, 7-point scale ranging from never to every day [1-7 points]),^18^ which asked how often clinicians felt emotionally drained by their work (7-point Likert scale ranging from never to every day). Clinicians were also asked whether they had made changes to the child’s treatment at the parent’s request although they felt it wasn’t in the child’s best interest (yes, often; yes, rarely; no).

### Statistical Analysis

For the Relationship Challenges Scale–Parent version, we considered relationships to be challenging if a parent responded to any single question in the 2 lowest of 4 possible categories, reflecting strategies used by existing patient experience surveys, which identify problem areas based on single item responses.^16,19,20,21^ For the clinician version, we considered challenges to be present if a clinician reported responses in the 3 lowest of 6 possible response categories to any question. Exploratory analyses used summary measures of all items for each scale with similar results.

Proportions of parents and clinicians reporting challenging relationships were calculated, and marginal asymmetry between parent and clinician perspectives for 338 paired assessments was determined using McNemar test. Bivariable and multivariable multilevel logistic regression analyses were used to evaluate factors associated with parent-identified and clinician-identified challenges, accounting for 1 to 2 relationships per parent and multiple possible relationships per clinician. Factors associated with parent-identified challenges were evaluated for 676 relationships with parent reports; factors associated with clinician-identified challenges were evaluated in 338 relationships with clinician reports. A sensitivity analysis limited parent assessments to 338 relationships with paired clinician surveys (eTable 1 in the Supplement). Covariates were dichotomized as specified in tables and text. In addition to parent and clinician race and ethnicity, we examined the association of a match between parent and clinician race and ethnicity with study outcomes. Parents with Asian or other race or ethnicity were combined for analysis owing to small sample sizes. Clinicians who were Asian, Black, or Hispanic were combined into a single category as non-White or Hispanic for analysis owing to small sample sizes. For health care systems challenges, more teamwork was defined as other doctors and nurses involved in their child’s care working together well always or often. Mixed messages were considered uncommon when parents said they were told different things by different people rarely or never. Communication across transitions was considered consistent when parents reported that different doctors and nurses caring for the child were always or often aware of the child’s medical history. Patient-centeredness across transitions was considered to be high when different doctors and nurses caring for the child were aware of the child’s special needs always or often.

A backward selection procedure was used to construct the multivariable model, with a significance level for removal from the model of *P* ≥ .05. Parent sex and race and ethnicity were included in the parent model regardless of significance, as were clinician sex and role in the clinician model. After we identified variables for inclusion, we performed multiple imputation of missing data for covariates using chained equations. We created 5 imputed data sets and estimated model parameters for each. Results from imputed data sets were integrated by Rubin rules and reported as odds ratios (ORs) with 95% CIs.^22^

The proportions of relationships in which clinicians used strategies to work with parents were calculated, with bivariable multilevel logistic regression used to compare proportions in relationships with and without challenges, accounting for multiple relationships per clinician. Bivariable multilevel logistic regression was also used to evaluate potential outcomes of clinician-defined challenging relationships, including burnout and changes in treatment. Burnout was defined as a response of 5 to 7 on a 7-point scale; treatment changes were defined as responses of yes, often and yes, rarely.

Statistical analyses were conducted using R statistical software version 4.0.2 (R Project for Statistical Computing). *P* values were 2-sided, and statistical significance was set at *P* < .05. Data were analyzed from July 2020 to August 2021.

## Results

Of 515 eligible parents, 417 parents (80.9%) completed the survey, including 42 parents who completed the survey in Spanish, with 400 parents completing relevant questions on 676 parent-clinician relationships (ie, the study population). Among 129 eligible clinicians, 80 clinicians (62.0%) completed matched surveys (ie, the study population), corresponding to 273 parents (65.5% of parents who completed the survey) and 338 relationships.

Among the study population of 400 participating parents, there were 298 (74.5%) women, 25 Asian individuals (6.3%), 28 Black individuals (7.0%), 97 Hispanic individuals (24.3%), 223 White individuals (55.8%), and 10 individuals (2.4%) with other race or ethnicity; race and ethnicity data were missing for 17 (4.3%) individuals. Characteristics of all participating parents were similar to those of parents with matched clinician surveys (Table 1). Of the study population of 80 participating clinicians, there were 57 (71.3%) women, 15 Asian individuals (18.8%), 1 Black individual (1.2%), 2 Hispanic individuals (2.5%), 62 White individuals (77.5%), and 0 individuals with other race or ethnicity. Nearly half of clinicians (38 individuals [47.5%]) were attending physicians, while 32 clinicians (40.0%) were fellows and 10 clinicians (12.5%) were nurse practitioners (Table 2).

**Table 1.** Parent and Child Characteristics

Characteristic	Individuals, No. (%)	
	Participating parents (n = 400)	Parents with paired clinician surveys (n = 273)
**Parent characteristic**		
Sex		
Women	298 (74.5)	204 (74.7)
Men	97 (24.2)	67 (24.5)
Missing	5 (1.2)	2 (0.7)
Race and ethnicity		
Asian	25 (6.3)	18 (6.6)
Black	28 (7.0)	17 (6.2)
Hispanic	97 (24.2)	67 (24.5)
White	223 (55.8)	154 (56.4)
Other^a^	10 (2.4)	9 (3.3)
Missing	17 (4.3)	8 (2.9)
Age, y		
<30	64 (16.0)	42 (15.4)
30-39	171 (42.8)	119 (43.6)
40-49	111 (27.8)	78 (28.6)
≥50	45 (11.2)	30 (11.0)
Missing	9 (2.2)	4 (1.5)
Education		
≤High school graduate	87 (21.8)	58 (21.2)
Some college or technical school	85 (21.2)	62 (22.7)
College graduate	131 (32.8)	89 (32.6)
Graduate or professional school	92 (23.0)	62 (22.7)
Missing	5 (1.2)	2 (0.7)
Marital status		
Married or living as married	288 (72.0)	205 (75.1)
Not married and not living as married	108 (27.0)	67 (24.5)
Missing	4 (1.0)	1 (0.4)
Primary language spoken at home		
English	303 (75.8)	210 (76.9)
Spanish	12 (3.0)	7 (2.6)
Other	12 (3.0)	8 (2.9)
Bilingual (English and Spanish)	18 (4.5)	14 (5.1)
Bilingual (English and other)^b^	14 (3.5)	12 (4.4)
Missing	41 (10.2)	22 (8.1)
**Child characteristic**		
No.	400	273
Age, y		
<2	49 (12.2)	32 (11.7)
2-4	107 (26.8)	75 (27.5)
5-7	57 (14.2)	41 (15.0)
8-10	40 (10.0)	25 (9.2)
11-17	147 (36.8)	100 (36.6)
Cancer type		
Hematologic cancer	262 (65.5)	190 (69.6)
Brain or solid tumor	138 (34.5)	83 (30.4)

^a^ Given the small number in the other race and ethnicity category, these are not further defined to protect identities.

^b^ Participants were not asked to specify other language.

**Table 2.** Clinician Characteristics

Characteristic	Clinicians, No. (%) (N = 80)
Sex	
Women	57 (71.3)
Men	23 (28.7)
Race and ethnicity	
Asian	15 18.8)
Black	1 (1.2)
Hispanic	2 (2.5)
White	62 (77.5)
Other^a^	0
Clinician type	
Attending physician	38 (47.5)
Fellow	32 (40.0)
Nurse practitioner	10 (12.5)
Time in clinical care, hr/wk	
<10	10 (12.5)
10-19	24 (30.0)
≥20	45 (56.2)
Missing	1 (1.2)
Time since graduation from medical or nursing school, y	
<10	38 (47.5)
10-19	26 (32.5)
≥20	13 (16.2)
Missing	3 (3.8)

^a^ Given the small number in the other race and ethnicity category, these are not further defined to protect identities.

Among 338 relationships with paired parent and clinician surveys, 81 relationships (24.0%) were considered challenging by parents and 127 relationships (37.6%) were considered challenging by clinicians; 33 relationships (9.8%) were considered challenging by parent and clinician. When views were not aligned, clinicians reported more challenges than parents; in 48 relationships (14.2%), parents experienced challenges that were not reported by clinicians, whereas in 94 relationships (27.8%), clinicians experienced challenges not reported by parents (*P* < .001) (Table 3).

**Table 3.** Associations Between Parent and Clinician Perspectives

	Relationships, No. (%) (N = 338)^a^^,^^b^		
	Parent-reported challenges	Challenges by parent report	Row total
Clinician-reported challenges	33 (9.8)	94 (27.8)	127 (37.6)
Challenges by clinician report	48 (14.2)	163 (48.2)	211 (62.4)
Column total	81 (24.0)	257 (76.0)	338

^a^ Unique relationships with paired parent and clinician reports.

^b^ *P* < .001, McNemar test of symmetry.

In total, 114 parents (28.5%) reported at least 1 challenging relationship. More than half of clinicians (42 clinicians [52.5%]) had at least 1 relationship that a parent considered challenging, and 54 clinicians (67.5%) had at least 1 relationship that they considered challenging. In bivariable analyses, parents who were Asian or had other race or ethnicity (OR, 2.78; 95% CI, 1.19-6.52) or Hispanic (OR, 1.94; 95% CI, 1.06-3.56) were more likely to experience challenging relationships compared with White parents. Challenges among 676 relationships identified by parents were reported for 21 of 61 relationships in which the parents were Asian or had other race or ethnicity (34.4%) and 40 of 154 relationships in which the parents were Hispanic (26.0%) compared with 65 of 390 relationships in which the parents were White (16.7%). A greater percentage of relationships in which the parents were Black (12 of 46 parents [26.1%]) also had challenges compared with relationships in which the parents were White, although this difference was not statistically significant. Parents who had lower educational attainment (OR for high school or less vs more than high school, 2.49; 95% CI, 1.35-4.58) were also more likely to experience challenging relationships (reported for 46 of 138 relationships in which parents had high school education or less [33.3%] vs 100 of 531 relationships in which parents had more than high school education [18.8%]), as were parents with anxiety (OR, 2.39; 95% CI, 1.39-4.13) or depression (OR, 2.70; 95% CI, 1.58-4.61) (Table 4). In addition, health care systems issues, including less interdisciplinary teamwork (OR vs more teamwork, 3.55; 95% CI, 1.34-9.39), mixed messages common (OR vs mixed messages uncommon, 5.08; 95% CI, 2.61-9.92), inconsistent communication across transitions (OR vs consistent communication, 4.91; 95% CI, 2.57-9.41), and less patient-centeredness across transitions (OR vs more patient-centeredness, 8.97; 95% CI, 3.39-23.71) were associated with increased likelihood of parent-defined challenges. In a multivariable model adjusted for parent sex, Asian or other race or ethnicity (OR vs White race, 3.62; 95% CI, 1.59-8.26), lower education (OR for high school or less vs more than high school, 3.03; 95% CI, 1.56-5.90), anxiety (OR, 2.14; 95% CI, 1.27-3.61), mixed messages common vs uncommon (OR, 4.42; 95% CI, 2.21-8.83), and less patient-centeredness across transitions (OR vs more patient-centeredness, 5.84; 95% CI, 2.21-15.38) were associated with parent-identified challenges (Table 5).

**Table 4.** Bivariable Analyses of Factors Associated With Relationship Challenges

Factor	Relationships with parent-defined challenges, No. (%)^a^	OR (95% CI)	Relationships with clinician-defined challenges, No. (%)^b^	OR (95% CI)
**Parent factor**				
Sex				
Women	107/516 (20.7)	0.84 (0.46-1.53)	94/253 (37.2)	1 [Reference]
Men	37/152 (24.3)	1 [Reference]	30/82 (36.6)	0.89 (0.51-1.54)
Race or ethnicity				
Asian or other^c^^,^^d^	21/61 (34.4)	2.78 (1.19-6.52)	16/36 (44.4)	1.28 (0.61-2.69)
Black	12/46 (26.1)	1.83 (0.68-4.89)	6/19 (31.6)	0.72 (0.25-2.03)
Hispanic	40/154 (26.0)	1.94 (1.06-3.56)	24/77 (31.2)	0.72 (0.40-1.28)
White	65/390 (16.7)	1 [Reference]	76/196 (38.8)	1 [Reference]
Education				
≤High school	46/138 (33.3)	2.49 (1.35-4.58)	29/71 (40.8)	1.23 (0.69-2.22)
>High school	100/531 (18.8)	1 [Reference]	96/264 (36.4)	1 [Reference]
Primary language				
English	104/521 (20.0)	1 [Reference]	93/262 (35.5)	1 [Reference]
Other^e^	25/93 (26.9)	1.56 (0.72-3.35)	22/48 (45.8)	1.53 (0.80-2.93)
Anxiety score				
Not suggestive of anxiety	51/333 (15.3)	1 [Reference]	52/158 (32.9)	1 [Reference]
Suggestive of anxiety	91/317 (28.7)	2.39 (1.39-4.13)	70/168 (41.7)	1.41 (0.88-2.26)
Depression score				
Not suggestive of depression	66/417 (15.8)	1 [Reference]	73/210 (34.8)	1 [Reference]
Suggestive of depression	74/230 (32.2)	2.70 (1.58-4.61)	47/117 (40.2)	1.20 (0.73-1.96)
**Child factor**				
Age, y				
<2	20/83 (24.1)	1 [Reference]	15/42 (35.7)	1 [Reference]
2-4	38/177 (21.5)	0.92 (0.38-2.23)	33/94 (35.1)	1.65 (0.65-4.14)
5-7	23/95 (24.2)	1.11 (0.41-3.00)	20/46 (43.5)	1.16 (0.42-3.23)
8-10	18/71 (25.4)	1.19 (0.41-3.46)	11/32 (34.4)	1.21 (0.56-2.64)
11-17	48/250 (19.2)	0.80 (0.34-1.89)	48/124 (38.7)	1.59 (0.90-2.79)
Cancer type				
Hematologic cancer	107/442 (24.2)	1 [Reference]	79/231 (34.2)	1 [Reference]
Solid or brain tumor	40/234(17.1)	0.59 (0.33-1.04)	48/107 (44.9)	1.59 (0.90-2.79)
**Clinician factor**				
Role				
Attending physician	75/351 (21.4)	1 [Reference]	66/182 (36.3)	1 [Reference]
Fellow physician	39/192 (20.3)	0.99 (0.60-1.66)	37/94 (39.4)	0.99 (0.51-1.92)
Nurse practitioner	29/104 (27.9)	1.57 (0.85-2.89)	24/62 (38.7)	1.08 (0.46-2.50)
Sex				
Women	105/486 (21.6)	1 [Reference]	100/258 (38.8)	1 [Reference]
Men	37/161 (23.0)	1.17 (0.69-1.99)	27/80 (33.8)	0.75 (0.46-1.49)
Race and ethnicity				
White	116/521 (22.3)	1 [Reference]	108/286 (37.8)	1 [Reference]
Nonwhite or Hispanic^f^	26/126 (20.6)	0.85 (0.46-1.57)	19/52 (36.5)	0.86 (0.40-1.88)
Time in practice, y				
<10	35/228 (15.4)	1 [Reference]	37/120 (30.8)	1 [Reference]
10-19	45/209 (22.0)	0.93 (0.43-2.04)	57/113 (50.4)	2.58 (1.32-5.05)
≥20	37/170 (22.0)	0.94 (0.05-1.92)	30/93 (32.3)	1.13 (0.52-2.44)
Parent-clinician racial and ethnic match				
Matched	58/321 (18.1)	1 [Reference]	69/181 (38.1)	1 [Reference]
Unmatched	77/301 (25.8)	1.56 (0.95-2.57)	53/147 (36.1)	0.88 (0.54-1.46)
Systems of care				
Interdisciplinary teamwork^g^				
More teamwork	128/630 (20.4)	1 [Reference]	110/311 (35.4)	1 [Reference]
Less teamwork	17/39 (43.6)	3.55 (1.34-9.39)	14/23 (60.9)	2.67 (1.08-6.62)
Mixed messages^h^				
Mixed messages uncommon	103/578 (17.8)	1 [Reference]	103/290 (35.5)	1 [Reference]
Mixed messages common	42/86 (48.8)	5.08 (2.61-9.92)	20/42 (47.6)	1.59 (0.81-3.13)
Communication across transitions^i^				
Consistent	101/575 (17.6)	1 [Reference]	104/285 (36.5)	1 [Reference]
Inconsistent	44/92 (47.8)	4.91 (2.57-9.41)	21/49 (42.9)	1.36 (0.71-2.61)
Patient-centeredness across transitions^j^				
More patient-centered	121/630 (19.2)	1 [Reference]	117/316 (37.0)	1 [Reference]
Less patient-centered	25/41 (61.0)	8.97 (3.39-23.71)	8/20 (40.0)	1.07 (0.41-2.82)

Abbreviation: OR, odds ratio.

^a^ For parent-defined challenges, there were 676 relationships with parent reports. Percentages are out of the total number of relationships for each factor among these 676 relationships.

^b^ For clinician-defined challenges, there were 338 relationships with clinician reports. Percentages are out of the total number of relationships for each factor among these 338 relationships.

^c^ Given the small number in the other race and ethnicity category, these are not further defined to protect identities.

^d^ Asian and other race or ethnicity were combined owing to small cell sizes, which precluded separate analyses.

^e^ Participants were not asked to specify other language.

^f^ Included groups are Asian, Black, and Hispanic individuals. Groups were combined owing to the small sample size of these groups.

^g^ More teamwork was defined as other doctors and nurses involved in the child’s care working together well always or often.

^h^ Mixed messages were considered uncommon when parents said they were told different things by different people rarely or never.

^i^ Communication across transitions was considered consistent when parents reported that different doctors and nurses caring for the child were always or often aware of the child’s medical history.

^j^ Patient-centeredness across transitions was considered to be high when different doctors and nurses caring for the child were aware of the child’s special needs always or often.

**Table 5.** Multivariable Analysis of Factors Associated With Relationship Challenges Identified by Parents

Factor	Odds Ratio (95% CI)^a^
Parent sex	
Women	1 [Reference]
Men	0.73 (0.38-1.38)
Parent race and ethnicity	
Asian or other^b^	3.62 (1.59-8.26)
Black	1.27 (0.46-3.49)
Hispanic	1.60 (0.83-3.10)
White	1 [Reference]
Parent education	
>High school	1 [Reference]
≤High school	3.03 (1.56-5.90)
Parent anxiety score^c^	
Not suggestive of anxiety	1 [Reference]
Suggestive of anxiety	2.14 (1.27-3.61)
Mixed messages^d^	
Mixed messages uncommon	1 [Reference]
Mixed messages common	4.42 (2.21-8.83)
Patient-centeredness across transitions^e^	
More patient-centered	1 [Reference]
Less patient-centered	5.84 (2.21-15.38)

^a^ There were 676 relationships with parent reports. Analyses were adjusted for parent sex regardless of significance.

^b^ Other included Native American individuals, individuals with mixed race or ethnicity, and individuals with other race or ethnicity. Mixed race or ethnicity was reported by the participant as a written response, while other 2 categories were available as choices.

^c^ The Hospital Anxiety and Depression Scale was used to evaluate depression and anxiety, with scores of 8 or more on each subscale considered suggestive of the condition.

^d^ Mixed messages were considered uncommon when parents said they were told different things by different people rarely or never.

^e^ Patient-centeredness across transitions was considered to be high when different doctors and nurses caring for the child were aware of the child’s special needs always or often.

No parent or child attributes were associated with clinician-reported challenges. Clinicians who had been in practice 10 to 19 years were more likely to report challenging relationships than those who had been in practice less than 10 years, in bivariable analysis (OR, 2.58; 95% CI, 1.32-5.05) (Table 4) and multivariable analysis (OR, 2.65; 95% CI, 1.30-5.43) (eTable 2 in the Supplement) after adjustment for clinician sex and role. Less interdisciplinary teamwork, as reported by parents, was associated with more clinician-defined challenges (OR vs more interdisciplinary teamwork, 2.67; 95% CI, 1.08-6.62) in bivariable analysis (Table 4) but not multivariable analysis.

Clinicians reported using a variety of strategies to work with parents. Most such strategies were used more often in 127 relationships in which clinicians perceived challenges vs 211 relationships in which clinicians did not perceive challenges, including holding regular family meetings (22 relationships [17.3%] vs 33 relationships [6.2%]; *P* = .009), setting limits or boundaries (26 relationships [20.5%] vs 4 relationships [1.9%]; *P* < .001), giving extra time and attention (66 relationships [52%] vs 60 relationships [28.4%]; *P* < .001), and seeking advice from another clinician (from a physician or nurse practitioner: 26 relationships [20.5%] vs 11 relationships [5.2%]; *P* < .001; from a nurse: 17 relationships [13.4%] vs 5 relationships [2.4%]; *P* = .002; and from a psychosocial clinician: 33 relationships [26%] vs 18 relationships [8.5%]; *P* < .001) (Figure, part A). In contrast, no strategies were used by clinicians more often in relationships in which parents experienced challenges vs when parents did not experience this (Figure, part B).

**Figure.**
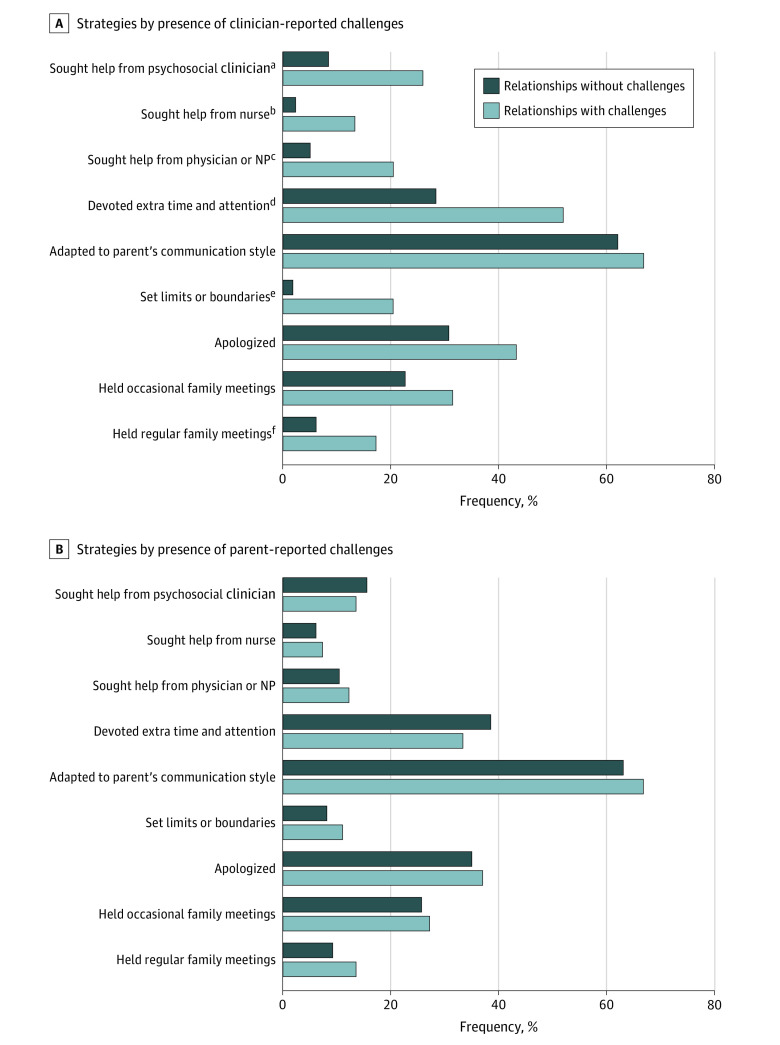
Strategies Used by Clinicians to Work With Parents NP indicates nurse practitioner. ^a^*P* < .001. ^b^*P* < .001. ^c^*P* < .001. ^d^*P* < .001. ^e^*P* < .001. ^f^*P* = .002.

Burnout was more common among clinicians who reported challenging relationships (OR, 2.37; 95% CI, 1.34-4.25). Clinicians who reported challenges were also more likely to report that they had made changes to the child’s treatment that they worried were not in the child’s best interests (OR, 14.98; 95% CI, 9.01-20.95), although such reports were rare, occurring among 9 clinicians.

## Discussion

The parent-clinician relationship is a core aspect of high-quality patient-centered and family-centered care in pediatric oncology. A successful therapeutic relationship offers a better experience of care for patients and families and a place to return for information, complex decisions, and support. Clinicians also stand to benefit from better therapeutic relationships, because challenges can be associated with stress and burnout. In this survey study of parents and clinicians of children with cancer treated at 2 centers, nearly one-quarter of early therapeutic relationships, soon after diagnosis, were considered challenging by parents and more than one-third were considered challenging by clinicians.

Strikingly, parent and clinician experiences were not well aligned; approximately 1 in 10 relationships were characterized as challenging by parent and clinician. While this may, in part, be associated with different definitions and scales used for parents vs clinicians, it also highlights the different experiences that parents and clinicians can have in the same relationship. In addition, while clinicians used strategies to try to work with parents successfully, strategies were used most often in relationships the clinicians perceived as challenging and not in relationships in which parents felt poorly served. This mismatch suggests an opportunity to better identify and respond to parent experiences in order to improve the family-centeredness of care.

While our study does not allow us to identify specific avenues to repair or prevent challenges, our findings suggest that one important step may be increasing clinician awareness of parental dissatisfaction when it occurs. Early identification of challenges, through routine assessment or efforts to help parents self-advocate for more productive relationships, may help clinicians to respond to relationship challenges and improve experiences for parents. This possibility suggests opportunities for future research.

Notably, parent-reported challenges were disproportionately borne by parents who were members of minority racial and ethnic groups and had lower educational attainment. This includes parents who were Asian or had other race or ethnicity, 34.4% of whose relationships included challenges. While not statistically significant in multivariable analyses, Black and Hispanic parents each experienced challenges in more than one-quarter of their relationships, compared with 16.7% of relationships among White parents. In addition, one third of relationships among parents whose education stopped at high school involved challenges. These findings fit with larger patterns of care disparities, including communication disparities, in oncology.^23,24,25,26,27,28,29,30,31,32,33^ Efforts to understand these differences more deeply and respond with focused efforts at improvement are critical so that all families have access to high-quality patient-centered and family-centered care. We examined racial and ethnic match between parents and clinicians as a potential mitigating factor^34^ but did not identify an association in our sample, which had limited clinician diversity. Nonetheless, enhanced diversity of the workforce remains an important goal for many reasons.^35,36^

Parents who experienced challenging relationships were also more likely to have symptoms of anxiety and depression. Our data cannot ascertain whether anxiety and depression were factors associated with challenges or outcomes of relationship challenges. Nonetheless, psychosocial care is a standard of care in pediatric oncology, and existing guidelines recommend early assessment of parental mental health needs with provision of appropriate interventions.^37,38,39^ Parents with psychological distress deserve support for many reasons, and this finding adds another.

We also found that wider health care systems issues, including mixed messages and a lack of patient-centeredness across care transitions, may be associated with exacerbation of challenges. When parents felt that other staff members were aware of their child’s unique needs, for example, they were less likely to identify relationships with their primary clinicians as challenging. Clinicians cannot be present at every encounter and cannot control the care in every setting, but our findings suggest that parents felt that negative experiences reflected poorly on their primary clinician. Focused efforts to improve systems may have the added benefit of strengthening relationships with clinicians who provide longer term clinical care.

Notably, most clinicians experienced challenging relationships, and clinicians who reported challenges were more likely to experience burnout, although whether this was a factor associated with challenges or an outcome of challenges is unknown. In rare cases, clinicians also reported altering the child’s care in response to parental wishes. These issues suggest that there are costs associated with challenges, including a potential association with worse patient care. However, while clinicians may experience feelings of failure over difficulties,^11^ most clinicians will experience these issues from time to time. Our findings suggest that we need to recognize this as a normal part of practice, even as we strive to improve care and experiences for all families.

### Limitations

This study has several limitations. These include clinician nonresponse, with clinician responses available for approximately two-thirds of parents, raising concerns for selection bias, as well as the potential that missing data impacted generalizability and interpretability of findings. In addition, the grouping of the small numbers of those who identified as “other racial and ethnic minority groups” with Asian respondents may have confounded the overall comparisons. Clinicians may additionally have declined permission for researchers to approach parents with whom relationships were challenging. We also used novel instruments to assess challenging relationships given a lack of existing instruments for pediatrics. This study lacked child perspectives, and we surveyed 1 parent per family, although relationships may differ between parents in the same family. We performed this work at 2 large academic cancer centers and were able to recruit a diverse parent population, but the numbers of Black parents were limited. There was limited clinician diversity. Future work may benefit from inclusion of smaller centers and ongoing efforts to include a diverse population.

## Conclusions

Parents of children with cancer enter relationships with oncology clinicians at one of the most vulnerable and challenging times in their lives. That so many parents are able to trust near-strangers with the lives of their children, and to quickly develop relationships of mutual respect and understanding, is remarkable. However, all parents deserve this experience. Attention to parental mental health, broader systems of care, and the needs of parents with lower educational attainment and who are members of racial and ethnic minority groups may help support the development of therapeutic relationships between parents and clinicians of children with cancer and other life-threatening illnesses.

## Notes

Supplement. **eAppendix 1.** Relationship Challenges Scale Parent Version **eAppendix 2.** Relationship Challenges Scale Clinician Version **eTable 1.** Bivariable Analyses of Factors Associated With Relationship Challenges Identified by Parents **eTable 2.** Multivariable Analysis of Factors Associated With Relationship Challenges Identified by Clinicians
