# Supplementary material for: Parent and Clinician Perspectives on Challenging Parent-Clinician Relationships in Pediatric Oncology
Source: JAMA Netw Open. 2021 Nov 17;4(11):e2132138. doi: 10.1001/jamanetworkopen.2021.32138 (PMC8600390; doi:10.1001/jamanetworkopen.2021.32138)
Supplement: Supplement. — eAppendix 1. Relationship Challenges Scale Parent Version eAppendix 2. Relationship Challenges Scale Clinician Version eTable 1. Bivariable Analyses of Factors Associated With Relationship Challenges Identified by Parents eTable 2. Multivariable Analysis of Factors Associated With Relationship Challenges Identified by Clinicians [file jamanetwopen-e2132138-s001.pdf]

Supplemental Online Content

Mack JW, Jaung T, Uno H, Brackett J. Parent and clinician perspectives on challenging parent-clinician relationships in pediatric oncology. *JAMA Netw Open*. 2021;4(11):e2132138.
doi:10.1001/jamanetworkopen.2021.32138

eAppendix 1. Relationship Challenges Scale Parent Version
eAppendix 2. Relationship Challenges Scale Clinician Version
eTable 1. Bivariable Analyses of Factors Associated With Relationship Challenges Identified by Parents
eTable 2. Multivariable Analysis of Factors Associated With Relationship Challenges Identified by Clinicians

This supplemental material has been provided by the authors to give readers additional information about their work.

© 2021 Mack JW et al. *JAMA Network Open.*

eAppendix 1. Relationship Challenges Scale Parent Version

Relationship Challenges Scale (Parent version).

How often would you say your child's oncology provider takes the time to listen to your concerns?

- ☐ Never
- ☐ At some office visits
- ☐ At most office visits
- ☐ At every office visit

How confident are you that your child's oncology provider is giving your child the best possible medical care?

- ☐ Not at all
- ☐ To a slight extent
- ☐ To some extent
- ☐ To a large extent

To what extent do you feel that your child's oncology provider is on your side?

- ☐ Not at all
- ☐ Somewhat
- ☐ Quite a bit
- ☐ A great deal

How much do you trust your child's oncology provider?

- ☐ Not at all
- ☐ Somewhat
- ☐ Quite a bit
- ☐ A great deal

How much do you feel your child's oncology provider cares about you and your child?

- ☐ Not at all
- ☐ Somewhat
- ☐ Quite a bit
- ☐ A great deal

How often do you feel that your child's oncology provider respects your opinion?

- ☐ Never
- ☐ Sometimes
- ☐ Most of the time
- ☐ Always

How often are you as involved as you want in making decisions about your child's care?

- ☐ Never
- ☐ Sometimes
- ☐ Most of the time
- ☐ Always

© 2021 Mack JW et al. *JAMA Network Open.*

How often is your child's oncology provider sensitive to your feelings?

- ☐ Never
- ☐ At some office visits
- ☐ At most office visits
- ☐ At every office visit

How often is your child's oncology provider sensitive to your child's feelings?

- ☐ Never
- ☐ At some office visits
- ☐ At most office visits
- ☐ At every office visit

How often do you feel you have to push your child's oncology provider to make sure your child is receiving the best possible medical care?

- ☐ Never
- ☐ At some office visits
- ☐ At most office visits
- ☐ At every office visit

How concerned are you that your child's oncology provider may make mistakes that could affect your child's care?

- ☐ Not concerned
- ☐ Slightly concerned
- ☐ Somewhat concerned
- ☐ Extremely concerned

© 2021 Mack JW et al. *JAMA Network Open.*

eAppendix 2. Relationship Challenges Scale Clinician Version

The following questions ask about your feelings about your communication and relationship with your patient's parents. **Please choose the one response that best represents how you feel.**

	Not at All					A Great Deal
How successful have you been in building a positive relationship with this patient's *parents*?	☐	☐	☐	☐	☐	☐
To what extent do you try to avoid or limit contact with this patient's parents when possible?	☐	☐	☐	☐	☐	☐
How time-consuming has it been to work with this patient's parents?	☐	☐	☐	☐	☐	☐
How challenging has it been to communicate with this patient's parents?	☐	☐	☐	☐	☐	☐
How frustrating have your interactions with this patient's parents been?	☐	☐	☐	☐	☐	☐
To what extent have interactions with this patient's parents made it difficult to provide the best possible medical care for the child?	☐	☐	☐	☐	☐	☐

© 2021 Mack JW et al. *JAMA Network Open.*

eTable 1. Bivariable Analyses of Factors Associated With Relationship Challenges Identified by Parents Limited to N=338 relationships with both parent and clinician reports.

Variable	% with Parent-Defined challenges	OR (95% CI)
**Parent factors**		
**Parent Gender**		
Female	22.9%	Ref
Male	25.6%	1.17 (0.63 -2.17)
**Parent Race**		
Asian/other	30.6%	1.82 (1.17-2.83)
Black	21.1%	1.08 (0.58-2.03)
Hispanic	28.6%	1.60 (1.15-2.23)
White	19.9%	Ref
**Parent Education**		
High school or less	38.0%	Ref
More than high school	20.1%	0.40 (0.21-0.75)
**Parent Primary Language**		
English	22.5%	Ref
Other	27.1%	1.27 (0.60-2.71)
**Parent Anxiety Score**		
Not suggestive of anxiety	18.4%	Ref
Suggestive of anxiety	30.4%	1.97 (1.13-3.45)
**Parent Depression Score**		
Not suggestive of depression	18.1%	Ref
Suggestive of depression	34.2%	2.40 (1.38-4.19)
**Child factors**		
**Child Age**		
<2 years	21.4%	Ref
2-4	25.5%	1.24 (0.49-3.17)
5-7	23.9%	1.13 (0.39-3.30)
8-10	31.6%	1.67 (0.54-5.23)
11-17	21.8%	1.00 (0.40-2.50)
**Child's Cancer Type**		
Hematologic Malignancy	26.4%	Ref
Solid or brain tumor	18.7%	0.64 (0.35-1.17)

© 2021 Mack JW et al. *JAMA Network Open.*

eTable 1, continued.

**Variable**	**% with Parent-Defined challenges**	**OR (95% CI)**
**Oncologist factors**		
**Role**		
Attending Physician	22.0%	Ref
Fellow Physician	23.4%	1.08 (0.58-1.99)
Nurse Practitioner	30.6%	1.53 (0.78-3.00)
**Gender**		
Female	24.0%	Ref
Male	23.8%	1.04 (0.56-1.92)
**Race**		
White	24.5%	Ref
Nonwhite/Hispanic	21.2%	0.83 (0.39-1.78)
**Years in practice**		
<10	28.9%	Ref
10-19	25.7%	1.02 (0.36-2.03)
20 or more	21.5%	0.82 (0.32-1.49)
**Parent-Oncologist racial/ethnic match**		
Matching race/ethnicity	20.4%	Ref
Mismatched race/ethnicity	26.5%	1.39 (0.80-2.41)
**Systems of care**		
**Interdisciplinary teamwork**		
Always/often/ sometimes	22.8%	Ref
Rarely/never	34.8%	1.85 (0.70-4.89)
**Mixed messages**		
Rarely/never	21.4%	Ref
Always/often/ sometimes	42.9%	2.83 (1.36-5.92)
**Communication across transitions**		
Always/often/ sometimes	21.4%	Ref
Rarely/never	40.8%	2.10 (1.30-5.27)
**Patient-centeredness across transitions**		
Always/often/ sometimes	21.5%	Ref
Rarely/never	65.0%	7.32 (2.54-21.12)

© 2021 Mack JW et al. *JAMA Network Open.*

eTable 2. Multivariable Analysis of Factors Associated With Relationship Challenges Identified by Clinicians

N=338 relationships with parent reports. Adjusted for clinician gender and role regardless of significance.

**Variable**	**Odds Ratio (95% CI)**
**Clinician gender**	
Female	Reference
Male	1.11 (0.51-2.39)
**Clinician Role**	
Physician	Reference
Nurse	1.05 (0.46-2.39)
**Years in practice**	
<10	Reference
10-19	2.65 (1.30-5.43)
>20	1.61 (0.50-2.69)

© 2021 Mack JW et al. *JAMA Network Open.*
